# Identification of fluorescence in situ hybridization assay markers for prediction of disease progression in prostate cancer patients on active surveillance

**DOI:** 10.1186/s12885-017-3910-4

**Published:** 2018-01-02

**Authors:** Katerina Pestova, Adam J. Koch, Charles P. Quesenberry, Jun Shan, Ying Zhang, Amethyst D. Leimpeter, Beth Blondin, Svetlana Sitailo, Lela Buckingham, Jing Du, Huixin Fei, Stephen K. Van Den Eeden

**Affiliations:** 1 Abbott Molecular, Inc., 1300 East Touhy Avenue, Des Plaines, IL 60018 USA; 2 Rush University Medical Center, Chicago, IL USA; 3 Kaiser Permanente Division of Research, Oakland, CA USA

**Keywords:** Prostate cancer, Genomic abnormalities, Prognosis, Risk stratification, FISH, Fluorescence in situ hybridisation, Biopsy

## Abstract

**Background:**

Prostate Cancer (PCa) is the second most prevalent cancer among U.S. males. In recent decades many men with low risk PCa have been over diagnosed and over treated. Given significant co-morbidities associated with definitive treatments, maximizing patient quality of life while recognizing early signs of aggressive disease is essential. There remains a need to better stratify newly diagnosed men according to the risk of disease progression, identifying, with high sensitivity and specificity, candidates for active surveillance versus intervention therapy. The objective of this study was to select fluorescence in situ hybridization (FISH) panels that differentiate non-progressive from progressive disease in patients with low and intermediate risk PCa.

**Methods:**

We performed a retrospective case-control study to evaluate FISH biomarkers on specimens from PCa patients with clinically localised disease (T1c-T2c) enrolled in Watchful waiting (WW)/Active Surveillance (AS). The patients were classified into cases (progressed to clinical intervention within 10 years), and controls (did not progress in 10 years). Receiver Operating Characteristic (ROC) curve analysis was performed to identify the best 3–5 probe combinations. FISH parameters were then combined with the clinical parameters ─ National Comprehensive Cancer Network (NNCN) risk categories ─ in the logistic regression model.

**Results:**

Seven combinations of FISH parameters with the highest sensitivity and specificity for discriminating cases from controls were selected based on the ROC curve analysis. In the logistic regression model, these combinations contributed significantly to the prediction of PCa outcome. The combination of NCCN risk categories and FISH was additive to the clinical parameters or FISH alone in the final model, with odds ratios of 5.1 to 7.0 for the likelihood of the FISH-positive patients in the intended population to develop disease progression, as compared to the FISH-negative group.

**Conclusions:**

Combinations of FISH parameters discriminating progressive from non-progressive PCa were selected based on ROC curve analysis. The combination of clinical parameters and FISH outperformed clinical parameters alone, and was complimentary to clinical parameters in the final model, demonstrating potential utility of multi-colour FISH panels as an auxiliary tool for PCa risk stratification. Further studies with larger cohorts are planned to confirm these findings.

**Electronic supplementary material:**

The online version of this article (doi:10.1186/s12885-017-3910-4) contains supplementary material, which is available to authorized users.

## Background

Prostate cancer (PCa) is the second most common cancer in men with approximately 161,360 men diagnosed annually in the US [1] and 1.1 million men worldwide [2]. Although the lifetime risk of developing PCa is approximately 1 in 6 (~16%), the risk of dying from the disease is only ~2% [3]. Early diagnosis and treatment improved survival in patients with high-risk cancers, however, concerns exist regarding over diagnosis and over treatment of men with lower-risk PCa due to co-morbidities and healthcare costs [4, 5]. Over the last 15–20 years, what was the watchful waiting (WW) approach has evolved into active surveillance (AS), and has gained popularity for managing lower-risk PCa [4, 6, 7]. Men on AS are monitored with periodic biopsies, prostate examination, and prostate-specific antigen (PSA) tests, and treated only when the PCa shows signs of progression.

Clinical parameters such as Gleason score, PSA levels, patient demographics, and combinations of these parameters are used to stratify patients with low-risk (indolent) prostate cancer for AS. Novel imaging and molecular diagnostic tools are emerging to aid in patient risk stratification and monitoring on AS [7–9]. New genomic biomarkers and biomarker panels, including gene copy number, rearrangements and germline mutations, are being assessed for association with clinically and histologically aggressive disease [10–12]. However, current methods still lack the precision needed to reliably discriminate men with varying PCa risks. Given that PCa is both a biologically and clinically heterogeneous disease that develops amidst diverse genetic and epigenetic changes [13–15], identification of molecular biomarkers that can reliably discriminate aggressive vs indolent disease, as well as biomarkers for monitoring of progression during AS is paramount.

Fluorescence in situ hybridisation represents a widely-used molecular technique that allows the detection of numerical and structural abnormalities in tissue and cytology specimens. Multiple chromosomal alterations have been reported in PCa, such as chromosome aneusomy, gain of the 8q24 (MYC) region, loss of 10q23 (PTEN) region, and translocations of ERG and ETV1 genes [13, 16–22].

In this study, we evaluated FISH biomarkers on a retrospective case-control cohort of 108 PCa patients on WW/AS in order to establish a panel that can differentiate non-aggressive prostate cancer from aggressive prostate cancer.

## Methods

### FISH probes

A total of 12 probes including 2 centromeric probes (CEP®) and 8 locus-specific identifiers (LSI®) were used. All probes were obtained from Abbott Molecular, Inc. (Des Plaines, IL). The probes were assembled in three four-color hybridisation probe mixes. Probe mix 1, consisted of SpectrumGold™ PTEN (10q23), SpectrumAqua™ CEP10 (10p11.1-q11.1), and a Dual Colour ERG Break-Apart probe containing SpectrumRed™ ERG Cen (21q22) and SpectrumGreen™ ERG Tel (21q22). Probe mix 2 included SpectrumGold™ NKX3.1 (8p21), SpectrumAqua™ CEP8 (8p11.1-q11.1), SpectrumRed™ FGFR1 (8p12) and SpectrumGreen™ MYC (8q24). Probe mix 3 contained SpectrumGold™ CDKN1B (9p21), SpectrumAqua™ NMYC (2p24), and the Dual Colour ETV1 Break-Apart probe containing SpectrumGreen™ ETV1 Cen (7p21) and SpectrumRed™ ETV1 Tel (7p21) probes. Additional probes, SpectrumAqua™ MDM2 (20q13.2) and SpectrumRed™ AURKA (20q13.2) were used in the initial feasibility study.

### Initial feasibility study on radical prostatectomy specimens

Fifty-two archived, formalin-fixed paraffin embedded (FFPE) radical prostatectomy (RP) specimens from patients with adenocarcinoma of prostate were collected at Rush Medical Center (RUMC), Chicago, IL. The specimen set included 10 patients with Gleason score of <6, 14 patients with Gleason score of 6, 19 patients with Gleason score of 7, and 9 patients with Gleason score of 8 and 9. Patient age ranged from 46 to 76 years old, with a median age of 62. The specimens were collected during the period from 1990 to 2012, with a follow up time of 4–15 years, with a median follow up time of 12.5 years. Thirty-two of the 52 patients recurred within 5 years (PSA progression or death of disease), and 20 remained disease-free with 8 to 15 years.

### Developmental study on prostate biopsy specimens

To further develop the assay, a study was conducted on core needle biopsy specimens collected by Kaiser Permanente Northern California (KPNC). The nested case-control included men with local stage prostate cancer who were classified as Very Low, Low or Intermediate risk disease, who had a diagnostic PSA level of 10 or under and a biopsy Gleason score of 7 or under and were part of an active surveillance program. “Cases” were men diagnosed with localised prostate cancer who had definitive evidence of disease progression within 10 years of diagnosis of prostate cancer. “Controls” were individuals matched to cases on age (+/− 10 years), disease stage and grade, PSA level, age, race, and dates of diagnosis and follow-up. Summary of primary clinical characteristics for cases and controls is provided in the Additional file 1. One hundred eighteen de-identified, blinded Formalin-Fixed Paraffin-Embedded (FFPE) tumour samples were received from the KPNC Biospecimen repository. The specimens were from the initial diagnostic biopsy, collected from 1997 to 2003. Each case had a minimum of 6 cores.

Specimens were from patients that either (1) have a minimum of 10 years follow-up data and did not show disease progression, or (2) had progression of disease within 10 years of diagnosis. Median follow up time for the patients on study was 13 years (11 years for the 41 patients who died, and 14 years for those patients who were alive at the time of the study initiation). Progressive disease was defined as showing progression to metastases confirmed by imaging or as three consecutive rises in PSA level during surveillance leading to definitive therapy. Of the patients with progressive disease, 25% progressed within 1 year, 50% progressed within 1 to 3 years, and 25% had a progression time of greater than 3 years.

The FFPE blocks were sectioned into a minimum of five 5-micron sections and applied to positively-charged microscope slides. The specimens were characterised by staining one out of 5–10 serial sections with haematoxylin and eosin (H&E) followed by examination by an expert pathologist at a central laboratory to mark (scribe) the tumour area and to assign Gleason scores following current grading criteria. The specimen slides used for the FISH assay procedure were within 10 serial sections of the respective H&E-stained slide to assure minimal separation of the areas examined by FISH from the areas evaluated by histopathology.

### Histological sample pretreatment and hybridisation

FFPE histological specimen slides were baked at 56 °C for 2–24 h and treated three times in Hemo-De (Scientific Safety Solvents) for 5 min each at room temperature, followed by two 1-min rinses in 100% ethanol at room temperature. Slides were then pretreated using Vysis IntelliFISH Universal FFPE Tissue Pretreatment and Wash Reagents as follows. Slides were incubated in pretreatment solution at 80 °C for 35 min, rinsed for 3 min in deionised water, incubated 10–20 min in 0.15% pepsin in 0.1 N HCl solution at 37 °C, and rinsed again for 3 min in deionized water. Slides then were dehydrated for 1 min each in 70, 85, and 100% ethanol and air-dried. Batch processing of slides was carried out in the VP 2000 Slide Processor (Abbott Molecular). After pretreatment, three slides from each specimen were hybridised with three hybridisation probe mixes containing FISH probes combined with blocking DNAs and LSI/WCP Hybridisation Buffer (Abbott Molecular, Inc., Des Plaines, IL). Ten microliters of each hybridisation probe mix were added to a specimen, a coverslip was applied and sealed with rubber cement. Slides and probes were co-denatured for 5 min at 73 °C and hybridised for 16–24 h at 37 °C on a ThermoBrite® Hybridisation System (Abbott Molecular, Inc.). After hybridisation, coverslips were removed by soaking the slides in 2X SSC/0.3% NP-40 for 2–5 min at room temperature, followed by a wash in 2X SSC/0.3% NP-40 at 73 °C for 2 min. The slides were then allowed to dry in the dark. Ten microliters of 4′,6-diamidino-2-phenylindole counterstain/antifade solution (DAPI I, Abbott Molecular, Des Plaines, IL) was added to the specimen, and a coverslip was placed on the slide prior to evaluation.

### FISH signal evaluation

The specimens were analysed using a fluorescence microscope equipped with single bandpass filters (Abbott Molecular, Des Plaines, IL) specific for DAPI, Spectrum Gold™, SpectrumRed™, SpectrumGreen™, and SpectrumAqua™. In addition, a dual bandpass Red/Green filter was used to evaluate break-apart ERG and ETV1 probes. For each specimen, 100 consecutive non-overlapped, intact interphase nuclei within the scribed area were enumerated.

### Statistical analysis

The following FISH parameters were calculated for the abnormal patterns of each probe, based on signal enumeration results:

- “Gain” percent cells with >2 signals;
- “Loss”, percent cells with <2 signals;
- “Homozygous” deletion – percent of cells with 0 FISH signals for a probe;
- “Ratio” – ratio of the average number of probe signals per cell to the average number of signals for the CEP control probe located on the same chromosome;
- “Split” – for a break-apart probe, green and red signals separated by a distance of ≥1 signal width: translocation detected;
- “2Edel” – for the ERG break-apart probe: separated green and red signals associated with the gain or amplification of single red signals and the concurrent loss of at least one of the green signals [23].

For the initial feasibility study, candidate probes and multicolour probe combinations were prioritised using ROC analysis and the Cox Proportional Hazards model, using disease recurrence or death from disease within the follow up period of 15 years (progression) as the outcome.

For the developmental study on the prostate biopsy specimens, “Cases” were designated as those patients who did not receive any curative treatment within 1 year of diagnosis but were classified as having progressive prostate cancer within 10 years of diagnosis, and “Controls” as those who did not receive curative or palliative treatment within 10 years of diagnosis and did not have evidence of progressive prostate cancer. The receiver operating characteristic (ROC) method [24] and correlation analysis were used to select and prioritise individual candidate FISH parameters. Individual FISH parameters were grouped in combinations, and the ROC method was used to (i) select optimal FISH parameter combinations by calculating and comparing the Area Under the Curve (AUC); (ii) select the optimal cut-off value for individual FISH probes by calculating and comparing the Distance From Ideal (DFI). AUC was used as the criterion for selecting the optimal FISH parameter combinations in respect to their ability to distinguish progressive (Case) vs. non-progressive disease (Control).

For each FISH parameter, cut-offs were established in a combinatorial analysis based on percentage of cells containing a genomic abnormality. Each cut-off was determined by simulating all possible cut-off combinations (for each parameter in the parameter combination), and choosing those cut-offs for each parameter that resulted in the lowest DFI for the parameter combination which provided both highest sensitivity and specificity. DFI is defined as $\sqrt{{\left(1-\mathrm{sensitivity}\right)}^2+{\left(1-\mathrm{specificity}\right)}^2}$. DFI represents the minimum distance from the ROC curve to the value of a sensitivity of 1 and a false positive rate (1-specificity) of 0. The DFI ranges from 0 to 1, with 0 being the ideal. In this analysis, FISH positivity and negativity was assigned based on the cut-off values, such that if any of the FISH parameters in the combination was greater than or equal to the cut-off, the specimen was considered positive, while if all FISH parameters in the combination were below the cut-off, the specimen was considered negative.

To evaluate the strength of the association between FISH parameters, clinical parameters and the progressive PCa, a logistic regression analysis was performed by using the selected probe sets and NCCN Prostate Cancer Risk Groups (“NCCN Risk Groups”). The Risk Groups are based on tumour stage, PSA, Gleason score and metastatic status and include Very Low, Low, Intermediate, High, Very High and Metastatic groups [25]. In this regression analysis, FISH parameters were treated as categorical variables based on optimal cut-offs from the AUC analysis. To determine if there was any significant correlation between individual FISH biomarker and the clinical parameters, Pearson’s correlation coefficients were calculated. A *p*-value less than 0.05 was considered to be statistically significant.

All analyses were performed using SAS version 9.2 or above (SAS Institute Inc., Cary, NC, USA.) by Abbott Molecular Biostatistics and Data Management Group.

## Results

### Initial feasibility – probe selection on radical prostatectomy specimens

FISH probes for this study were chosen based on the initial feasibility of multi-colour FISH on 52 formalin-fixed paraffin embedded RP specimens from patients with adenocarcinoma of prostate, collected at Rush Medical Center (RUMC), Chicago, IL. In the initial feasibility study, specimens were tested with 14 FISH probes: PTEN (10q23), NKX3.1 (8p21), CDKN1B (9p21), CEP10 (10p11.1-q11.1), MYC (8q24), AURKA (20q13.2), ERG Cen (21q22), ERG Tel (21q22), ETV1 Tel (7p21), ETV1 Cen (7p21), MDM2 (12q14-15), NMYC (2p24), FGFR1 (8p12), and CEP8 (8p11.1-q11.1). Candidate probes and multicolour probe combinations were prioritised using ROC analysis and Cox Proportional Hazards model, using disease recurrence or death of disease (DOD) within the follow up period of 15 years as the outcome. Analysis of probes and probe combinations demonstrated that grouping of complimentary biomarkers was needed to achieve maximum performance, and that combinations could be selected with the potential to predict longer progression free time for FISH test negative patients. Specifically, patients positive for either FISH parameter in the combination, had more risk of developing progression comparing to patients in the FISH (−) group with a Hazard Ratio (HR) of 4.65. Based on the initial feasibility study, probes that did not demonstrate prognostic value either alone, or in combinations, were eliminated, resulting in selection of the following probes for further testing on the prostate biopsy specimens from the AS cohort: PTEN, CEP10, ERG Cen, ERG Tel, NKX3.1, CEP8, FGFR1, MYC, CDKN1B, NMYC, ETV1 Cen and ETV1 Tel.

### Detection of cytogenetic abnormalities by FISH in prostate biopsy specimens

**Fig. 1:**
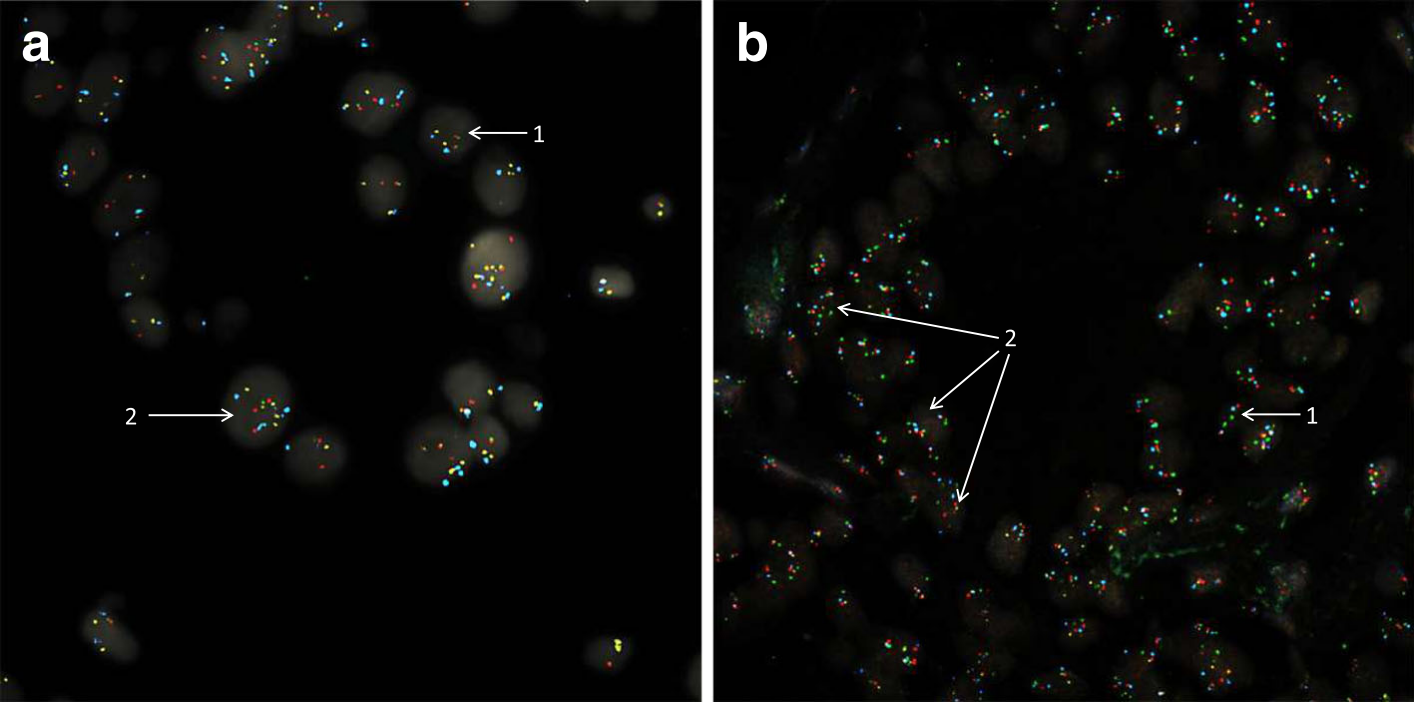
Example Images of Abnormal FISH Signals in Prostate Biopsy Tissue. **a** 4-colour probes set consisting of ERG (SpectrumRed/SpectrumGreen), PTEN (SpectrumGold) and CEP 10 (SpectrumAqua). Arrows indicate: 1, normal diploid cell; 2, translocation of ERG (2 Edel) shown by separation of red and green signals with an increased number of the individual red signals. **b** 4-colour probes set consisting of NKX3.1(SpectrumGreen), CEP 8 (SpectrumAqua), FGFR1 (SpectrumRed), and MYC(SpectrumGreen). Arrows indicate: 1, normal diploid cell; 2, cells displaying gain of copy numbers (>2)

A total of 118 specimens from KPNC with tumour area marked by a pathologist were pretreated and hybridised with each of the 3 multi-colour FISH probe sets. Of these specimens, 108 resulted in successful hybridisation (Fig. 1). The unsuccessful specimens did not withstand tissue pretreatment and the hybridisation process, demonstrated cell loss and lack of fluorescent signal, and could not be recovered with conventional troubleshooting methods. The reason for failures is likely attributable to the condition of a given specimen and variability in tissue fixation methods in the archived specimens.

No significant aneuploidy was observed in the specimens overall, with average copy numbers for the centromere probes CEP 8 and CEP 10 of 1.84 and 1.87, respectively. The value of less than 2 reflects typical truncation artefacts in FFPE tissue sections, and is expected. There was a slight increase in average copy number for CEP 8 and CEP 10 in cases as compared to controls (1.91 vs 1.80 for CEP 8 and 1.93 vs 1.82 for CEP 10).

Upon signal enumeration for each probe, mean percent cells with FISH abnormalities (gain, loss, homozygous loss, homozygous loss, split and 2Edel) per specimen was compared between cases (progressive disease) and controls (non-progressive disease). Correlation analysis between FISH parameters and clinical parameters (age, Gleason score and PSA) indicated that the only statistically significant correlation observed was for the NKX3.1 probe. The NKX3.1 Loss parameter had a statistically significant correlation with the Gleason score, while NKX3.1 Ratio parameter had a significant correlation with the tumour stage (Additional file 2). Based on this observation, NKX3.1 was excluded from further analysis.

### Selection of optimal probe combinations

Logistic regression ─ ROC curve analysis ─ was conducted to prioritise individual FISH parameters derived from signal enumeration with respect to their ability to distinguish progressive vs non-progressive disease, as described in the Methods section. Seven parameters (PTEN Homozygous, MYC gain, FGFR1 Gain, NMYC Gain, ETV1 Split, PTEN Loss and ERG 2Edel) were selected (Additional file 1) and then grouped in all possible combinations of 3–6 parameters. ROC curve analysis was performed on these combinations of parameters to identify combinations that can discriminate cases ─ those who progressed within 10 years (sensitivity) vs controls ─ those who did not progress within 10 years (specificity), with maximum sensitivity and specificity as judged by the AUC and DFI. Cut-off values for each probe were selected in this analysis. The optimal cut-offs expressed as percent of cells with an abnormality were in the ranges of 2–15 for amplification probes, 10–20 for deletion probes, and 4–10 for break apart probes. Parameter combinations with the highest AUC are shown in Table 1. The individual FISH parameters were not included since they were inferior to the combinations. Since both 2Edel and ETV1 Split parameters rely on 2 FISH probes, the probe combinations presented in Table 1 require 3–6 FISH probes. Interestingly, increasing the number of parameters from 4 to 5 did not appear to increase the AUC.

**Table 1:** Selected 3, 4 and 5-parameter combinations with the lowest DFI and the highest AUC

# Probes	FISH Parameter 1	FISH Parameter 2	FISH Parameter 3	FISH Parameter 4	FISH Parameter 5	FISH Parameter 6	AUC	DFI (Minimum)
3	MYC Gain	PTEN Homozygous		FGFR1 Gain			0.71	0.43
3	MYC Gain	PTEN Homozygous	NMYC Gain				0.73	0.43
4	MYC Gain	PTEN Homozygous	NMYC Gain	FGFR1 Gain			0.73	0.45
4	MYC Gain	PTEN Homozygous				ETV1 Split	0.72	0.41
5	MYC Gain	PTEN Homozygous	NMYC Gain		ERG 2Edel		0.73	0.45
5	MYC Gain	PTEN Homozygous	NMYC Gain			ETV1 Split	0.72	0.42
6	MYC Gain	PTEN Homozygous	NMYC Gain	FGFR1 Gain	ERG 2Edel		0.73	0.46

### Performance of FISH with clinical parameters in the logistic regression model

Logistic regression analysis using proposed cut-offs demonstrated that the selected parameter combinations were significant in stratifying cases from controls. In the logistic regression analysis, FISH had a significant contribution to the prediction of PCa outcome (progression) with the highest Odds Ratio (OR) of 7.005 observed for the combination of 5 probes (4 parameters), as shown in Table 2. FISH parameters were independent of clinical parameters in the model.

**Table 2:** Logistic Regression Models of FISH Only and FISH with Clinical Parameters (NCCN Risk Groups). Odds ratios, the corresponding confidence limits (CL) and *p* values are provided for both predictor variables (FISH and NCCN Risk Groups, respectively) in the second model. If any of the FISH parameters in the combination is greater than or equal to the cut-off, the specimen is considered positive; if all FISH parameters in the combination are below the cut-off, the specimen is considered negative

FISH Parameter Combination	*n* Cases	*n* Controls	FISH Only Model				FISH With Clinical Parameters Model							
			Odds Ratio	95% Wald Lower CL	95% Wald Upper CL	*p* Value	FISH				NCCN Risk Groups			
							Odds Ratio	95% Wald Lower CL	95% Wald Upper CL	*p* Value	Odds Ratio	95% Wald Lower CL	95% Wald Upper CL	*p* Value
MYC Gain & FGFR1 Gain & PTEN Homozygous	58	49	5.125	2.288	13.308	0.0001	6.221	2.441	15.851	0.0001	4.149	1.339	12.851	0.0136
MYC Gain & PTEN Homozygous & NMYC Gain	61	46	5.631	2.343	14.248	0.0001	5.889	2.299	15.084	0.0002	3.761	1.216	11.635	0.0215
MYC Gain & PTEN Homozygous & NMYC Gain & FGFR1 Gain	55	52	5.647	2.395	12.583	<0.0001	7.005	2.835	17.306	<0.0001	5.141	1.632	16.190	0.0052
MYC Gain & PTEN Homozygous & ETV1 Split	58	49	6.125	2.657	14.119	<0.0001	6.402	2.666	15.374	<0.0001	3.910	1.272	12.021	0.0173
MYC Gain & PTEN Homozygous & NMYC Gain & 2 Edel	62	45	5.173	2.343	14.248	0.0001	5.889	2.299	15.084	0.0002	3.761	1.216	11.635	0.0215
MYC Gain & PTEN Homozygous & NMYC Gain & ETV1 Split	62	45	6.205	2.616	16.831	<0.0001	6.431	2.463	16.789	0.0001	3.429	1.107	10.621	0.0326
MYC Gain & PTEN Homozygous &NMYC Gain & FGFR1 Gain & 2 Edel	63	44	4.761	2.015	10.533	0.0003	5.065	2.110	12.160	0.0003	4.029	1.328	12.224	0.0138

Clinical information was available to apply NCCN risk stratification criteria for 107 out of 108 patients in this study. Out of 107 patients, 24 were classified as High risk, 29 were classified as Intermediate risk, and 54 as Low and Very Low risk by these criteria. To assess whether FISH could be additive to risk stratification using NCCN criteria, risk groups based on clinical parameters were added to the regression model. According to Table 2, the combination of clinical parameters and FISH outperformed FISH alone for all FISH probe combinations: the OR for FISH was stronger when adjusted for risk group, as compared to unadjusted. We would like to note that in our analysis, patient age did not prove to be significant in either of the logistic regression models. Combination of clinical parameters with FISH resulted in Odds Ratios of 5.1–7.0. Therefore, those patients who are risk-stratified according to NCCN guidelines and who are also FISH positive appear to be seven times more likely to develop progression than those who are FISH-negative. For comparison, in the logistic regression analysis model that included only clinical parameters without FISH, the Odds Ratios were calculated to be 3.690 and 0.965 for NCCN Risk Groups and age, respectively. Additionally, both clinical parameters and FISH predictor variables were significant in this model. Thus, FISH appears to be additive in its predictive value to clinical parameters.

To assess predictive power of FISH with respect to disease progression by risk category, the patients were stratified in 3 categories: lower risk (including Low and Very Low risk NCCN groups), intermediate risk (Intermediate risk NCCN group), and higher risk (High risk NCCN group), and logistic regression analysis was performed on each group for FISH combinations (Table 3). Although sample size was relatively low in this study, FISH was statistically significant in discrimination of progressive vs non-progressive disease in lower and intermediate risk categories. In this analysis, the highest OR was observed in the intermediate risk category.

**Table 3:** Logistic Analysis of FISH Marker Combinations by Risk Category

FISH Parameter Combination	Lower Risk				Intermediate Risk				Higher Risk			
	*n* Cases	*n* Controls	Odds Ratio	*p* Value	*n* Cases	*n* Controls	Odds Ratio	*p* Value	*n* Cases	*n* Controls	Odds Ratio	*p* Value
MYC Gain & FGFR1 Gain & PTEN Homozygous	29	25	6.017	0.0033	16	13	7.333	0.0192	13	11	3.143	0.2483
MYC Gain & PTEN Homozygous & NMYC Gain	29	25	6.017	0.0033	17	12	11.998	0.0082	15	9	2.000	0.4691
MYC Gain & PTEN Homozygous & NMYC Gain & FGFR1 Gain	28	26	4.886	0.0074	16	13	16.500	0.0035	11	13	6.250	0.1247
MYC Gain & PTEN Homozygous & ETV1 Split	29	25	6.017	0.0033	15	14	10.083	0.0082	14	10	4.000	0.1657
MYC Gain & PTEN Homozygous & NMYC Gain & 2 Edel	29	25	6.017	0.0033	18	11	8.999	0.0179	15	9	2.000	0.4691
MYC Gain & PTEN Homozygous & NMYC Gain & ETV1 Split	29	25	6.017	0.0033	17	12	11.998	0.0082	16	8	2.600	0.3253
MYC Gain & PTEN Homozygous & NMYC Gain & FGFR1 Gain & 2 Edel	29	25	6.017	0.0033	19	10	6.857	0.0369	15	9	2.000	0.4691

## Discussion

The natural history of prostate carcinoma is highly variable, and it can be difficult, using current methodologies, to distinguish between patients with aggressive PCa that causes rapid tumour progression and significant clinical outcomes, and patients with indolent PCa. [26]. Undiagnosed, primarily indolent, prostate cancer is a common incidental finding in elderly men at autopsy [27]. This has important implications for management of PCa patients. Prostate specific antigen screening, for example, allows detection of more cases of asymptomatic prostate cancer, however, some of these tumours may not be biologically malignant. Patients with such indolent tumours would have little benefit from medical intervention, in part due to the comorbidities resulting from intervention therapy, such as radical prostatectomy (RP), which remains a preferred option for treatment of apparently localised disease. Thus, overtreatment of low-risk prostate cancer, which still occurs frequently, has significant impact on patient quality of life and health-related costs [28]. Radical prostatectomy represents a worthwhile medical intervention for patients cured of a life-threatening disease, however, not for patients whose tumours are not biologically aggressive, or for those patients who are discovered to have metastases a few months after surgery. This highlights the necessity for discovery and validation of reliable molecular markers to predict the behaviour of individual carcinomas.

FISH is an established molecular platform widely used in single, dual, or multicolour format for the detection of numerical and structural genomic abnormalities [29, 30]. The advantage of multicolour FISH is that this relatively simple technique allows for assessment of several genomic markers simultaneously in the context of the tissue specimen, capturing both genomic and structural heterogeneity of the prostate cancer. With the advent of automation and imaging systems, as well as assay chemistry improvements to reduce time to result, multiplex detection of more than four colours on one tissue specimen slide in 1–2 days has become possible [30, 31].

This study assessed whether multicolour FISH could be used to predict progressive PCa. In the preliminary feasibility, radical prostatectomy specimens were used to select FISH probes capable of discriminating patients who would recur within a 15-year follow-up period from those who would not. The hypothesis was that the disease recurrence in radical prostatectomy patients may reflect an aggressive form of prostate adenocarcinoma, with underlying molecular mechanisms that may overlap with those that enhance disease progression in patients on active surveillance. Based on the feasibility results, 12 probes were selected with a potential to discriminate progressive disease. These probes were organised in 3 probe sets and tested on core needle biopsy specimens obtained from patients who were enrolled in Active Surveillance and had a minimum of 10 years follow-up data.

FISH evaluation parameters were derived from enumeration results for each probe, and individual parameters, as well as parameter combinations, were analysed to identify the best combinations capable of discriminating progressive from indolent disease in the AS cohort. Combinations of FISH parameters in this study were selected that were statistically significant in predicting PCa outcome (progressive vs non-progressive), with the highest performance observed in 4–5 parameter combinations. If used in a multicolour FISH assay, these combinations would require 4–6 FISH probes – a level of multiplexing that is can be achieved with automated imaging systems [32].

Current clinical management and risk stratification of localised prostate cancer for enrolment into AS is based upon several clinical parameters including tumour stage, tumour grade as measured by the Gleason score, and the level of PSA assessed at the time of diagnosis [33]. Although these tools undoubtedly have predictive value, detecting progressive disease in a patient considered or selected for AS remains a challenge. It has been shown that many clinically low-risk prostate cancer patients are upgraded to a more aggressive disease at prostatectomy [34, 35]. According to recent estimates, approximately one-third of the patients are reclassified or upgraded as having a higher risk for progression during AS based on annual surveillance biopsy results [36, 37]. On the other hand, there remains a considerable discrepancy in current AS selection criteria, with a notion that some of the criteria may be too strict, thus excluding some patients in whom expectant management would be appropriate and safe [34, 38]. Therefore, it is important to determine whether genomic tissue biomarkers, such as the multicolour FISH panels used in this study, could improve the accuracy of risk stratification when used in combination with the standard of practice clinical parameters for enrolment into the AS. In the logistic regression that combined clinical risk stratification parameters (NCCN Risk Groups) with FISH, FISH parameter combinations were complimentary to clinical parameters and contributed significantly to the prediction of PCa outcome (progressive vs non-progressive). The combination of clinical parameters and FISH outperformed clinical parameters or FISH alone, with a maximum odd ratio of 7.0 achieved in the final model, as compared to 6.2 for the FISH parameters alone. Importantly, multicolour FISH appeared to add most value to risk stratification in the Intermediate risk group, a group of patients that could benefit from improved selection criterial for AS to reduce overtreatment without compromising survival [39]. Although the specimen set tested in this study is relatively small, an encouraging odds ratios of up to 16.5 were achieved in this group. It appears therefore plausible that utilizing multicolour FISH biomarkers could add value if incorporated into the clinical decision making process.

The limitation of this study is in distinguishing patients with the true rapid progression of the disease vs those with aggressive cancer missed on the initial biopsy due to the sampling error. The latter is especially relevant to the studies on archived specimens, approximately half of which have been collected under the original sextant biopsy protocol. However, one of the advantages of FISH is that it allows assessment of genomic biomarkers in the context of the tumour heterogeneity. In our earlier studies, we demonstrated that cytogenetic abnormalities could be observed by FISH within regions of benign histology extending beyond histologically evident tumour margin, indicating a field cancerisation effect in prostate cancer [40]. This characteristic may be beneficial to reduce sampling error and consequently the risk of missing a higher-grade cancer on initial biopsy prior to enrolment in AS, and would need to be addressed in the future studies. Evaluation of the biomarker combinations presented here warrants an additional study to validate their prognostic utility.

## Conclusions

Combinations of FISH parameters capable of discriminating progressive from non-progressive disease were selected based on ROC curve analysis. Combination of clinical parameters with FISH demonstrated improved performance when compared to clinical parameters or to FISH alone. Additionally, FISH proved complimentary to clinical parameters (NCCN Risk Groups) in the final model, demonstrating the potential utility of multicolour FISH panels as an auxiliary tool for PCa risk stratification. Further studies with larger cohorts are planned to confirm these findings.

## Additional files

Additional file 1: Summary of Patient Information and FISH. (DOCX 15 kb)

Additional file 2: Correlation Analysis Between FISH Biomarkers and Clinical Parameters. (DOCX 23 kb)

## Abbreviations

AS: Active surveillance
AUC: Area Under the Curve
CEP: Chromosome Enumerator Probe
DFI: Distance From Ideal
FFPE: Formalin Fixed Paraffin Embedded
FISH: Fluorescence in situ hybridisation
LSI: Locus Specific Identifier
NCCN: National Comprehensive Cancer Network
PCa: Prostate Cancer
PSA: Prostate-specific antigen
ROC: Receiver Operating Characteristic curve
RP: Radical prostatectomy
WW: Watchful waiting
