# Supplementary material for: Identification of fluorescence in situ hybridization assay markers for prediction of disease progression in prostate cancer patients on active surveillance
Source: BMC Cancer. 2018 Jan 2;18:2. doi: 10.1186/s12885-017-3910-4 (PMC5749018; doi:10.1186/s12885-017-3910-4)
Supplement: Supplementary file 1 — Summary of Patient Information and FISH. (DOCX 15 kb) [file 12885_2017_3910_MOESM1_ESM.docx]

**Additional file 1**

**Summary of Patient Information and FISH.**

|  | **Case** | **Control** |
| --- | --- | --- |
| **Clinical Parameters**  Patients, n (%) | 57 (52.8) | 51 (47.2) |
| Follow-up time, years: Mean +-SD | 10.9±4.3 | 12.8±1.8 |
| Age, years: Mean +-SD | 66.7±8.3 | 68.1±7.3 |
| PSA, ng/ml: Mean+-SD | 6.0±1.7 | 6.2±2.1 |
| Time to Progression, Mean | 2.2 | N/A |
| Time to progression, Median (25%, 75%) | 1.5 (0.9, 3.1) | N/A |
| Gleason Score (central pathology review) |  |  |
| ≤6 | 27 | 33 |
| 7 | 12 | 11 |
| ≥8 | 18 | 6 |
| Not available |  | 1 |
| Clinical Stage |  |  |
| T1a | 1 | 1 |
| T1b | 0 | 2 |
| T1c | 32 | 26 |
| T1NOS | 1 | 0 |
| T2b | 2 | 5 |
| T2NOS | 21 | 17 |
| **FISH Positivity, Individual Parameters***  MYC Gain, n (%) | 39 (68.4) | 15 (29.4) |
| NMYC Gain, n (%) | 10 (17.5) | 6 (11.8) |
| PTEN Homozygous Loss, n (%) | 4 (7.0) | 1 (2.0) |
| FGFR1 Gain, n (%) | 29 (50.9) | 18 (35.3) |
| ETV1 Split, n (%) | 8 (14.0) | 4 (7.8) |
| ERG 2Edel, n (%) | 3 (5.2) | 2 (3.9) |

* Cutoffs for each parameter were established in the combinatorial analysis as described in Methods.
