# Supplementary material for: Identification of fluorescence in situ hybridization assay markers for prediction of disease progression in prostate cancer patients on active surveillance
Source: BMC Cancer. 2018 Jan 2;18:2. doi: 10.1186/s12885-017-3910-4 (PMC5749018; doi:10.1186/s12885-017-3910-4)
Supplement: Supplementary file 2 — Correlation Analysis Between FISH Biomarkers and Clinical Parameters. (DOCX 23 kb) [file 12885_2017_3910_MOESM2_ESM.docx]

**Additional file 2**

**Correlation Analysis Between FISH Biomarkers and Clinical Parameters**

| **Category** | **FISH Parameter** | **N** | **Mean** | **SD** | **R** | **p Value** |
| --- | --- | --- | --- | --- | --- | --- |
| 1. FISH Biomarker vs Age | NMYC Gain | 97 | 10.2 | 25.43 | 0.19 | 0.0644 |
|  | ETV1 Split | 97 | 2.0 | 7.05 | -0.16 | 0.1077 |
|  | p27 Loss | 97 | 24.4 | 22.89 | -0.02 | 0.8347 |
|  | PTEN Loss | 106 | 34.0 | 28.22 | -0.05 | 0.5908 |
|  | PTEN Homozygous | 106 | 4.2 | 19.32 | 0.07 | 0.4648 |
|  | ERG 2 Edel | 106 | 2.7 | 9.61 | 0.17 | 0.0749 |
|  | ERG Split | 106 | 37.7 | 35.01 | 0.02 | 0.8480 |
|  | MYC Gain | 107 | 17.6 | 28.60 | 0.07 | 0.4617 |
|  | NKX3.1 Ratio | 107 | 1.0 | 0.19 | -0.18 | 0.0575 |
|  | FGFR1 Gain | 107 | 10.9 | 21.78 | 0.02 | 0.8597 |
|  | FGFR1 Loss | 107 | 31.7 | 25.11 | 0.07 | 0.4456 |
|  | NKX3.1 Loss | 107 | 41.0 | 30.25 | 0.13 | 0.1994 |

|  |  |  |  |  |  |  |  |
| --- | --- | --- | --- | --- | --- | --- | --- |
| **Category** | | **FISH Parameter** | **N** | **Mean** | **SD** | **R** | **p Value** |
| 2. FISH Biomarker vs Gleason Score | | NMYC Gain | 97 | 10.2 | 25.43 | 0.07 | 0.5077 |
|  |  | ETV1 Split | 97 | 2.0 | 7.05 | -0.01 | 0.9048 |
|  |  | p27 Loss | 97 | 24.4 | 22.89 | -0.05 | 0.6072 |
|  |  | PTEN Loss | 106 | 34.0 | 28.22 | 0.13 | 0.1954 |
|  |  | PTEN Homozygous | 106 | 4.2 | 19.32 | 0.17 | 0.0835 |
|  |  | ERG 2 Edel | 106 | 2.7 | 9.61 | -0.09 | 0.3621 |
|  |  | ERG Split | 106 | 37.7 | 35.01 | -0.09 | 0.3608 |
|  |  | MYC Gain | 106 | 17.8 | 28.69 | 0.11 | 0.2461 |
|  |  | NKX3.1 Ratio | 106 | **0.9** | **0.19** | **-0.27** | **0.0059** |
|  |  | FGFR1 Gain | 106 | 11.0 | 21.85 | -0.07 | 0.5077 |
|  |  | FGFR1 Loss | 106 | 31.6 | 25.20 | 0.19 | 0.0550 |
|  |  | NKX3.1 Loss | 106 | **41.0** | **30.39** | **0.23** | **0.0163** |

| **Category** | **FISH Parameter** | **N** | **Mean** | **SD** | **R** | **p Value** |
| --- | --- | --- | --- | --- | --- | --- |
| 3. FISH Biomarker vs PSA | NMYC Gain | 97 | 10.2 | 25.43 | 0.02 | 0.8257 |
|  | ETV1 Split | 97 | 2.0 | 7.05 | -0.08 | 0.4193 |
|  | p27 Loss | 97 | 24.4 | 22.89 | 0.01 | 0.9008 |
|  | PTEN Loss | 106 | 34.0 | 28.22 | -0.01 | 0.9480 |
|  | PTEN Homozygous | 106 | 4.2 | 19.32 | -0.00 | 0.9815 |
|  | ERG 2 Edel | 106 | 2.7 | 9.61 | 0.17 | 0.0869 |
|  | ERG Split | 106 | 37.7 | 35.01 | 0.12 | 0.2245 |
|  | MYC Gain | 107 | 17.6 | 28.60 | -0.01 | 0.9355 |
|  | NKX3.1 Ratio | 107 | 1.0 | 0.19 | -0.12 | 0.2326 |
|  | FGFR1 Gain | 107 | 10.9 | 21.78 | -0.08 | 0.3969 |
|  | FGFR1 Loss | 107 | 31.7 | 25.11 | 0.18 | 0.0622 |
|  | NKX3.1 Loss | 107 | 41.0 | 30.25 | 0.16 | 0.1018 |
